# A corpus-based comparison of stance markers in materials science research article abstracts by Chinese and English L1 authors

**DOI:** 10.1371/journal.pone.0344331

**Published:** 2026-08-07

**Authors:** Shunuan Xie, Linxiang Guo, Wen Zhang

**Affiliations:** School of Foreign Language Education, Qingdao University, Qingdao, Shandong, China; Shandong University, CHINA

## Abstract

Our study investigated stance markers used in Materials Science research article abstracts by native Chinese-speaking (Chinese L1) and native English-speaking (English L1) researchers, adopting the widely recognized interactional metadiscourse framework. We constructed two comparable corpora, each comprising 501 abstracts from research articles published between 2023 and 2025 in Materials Science journals indexed in the Web of Science Core Collection. Quantitative analysis revealed that there were significant cross-cultural differences between the two corpora: Chinese L1 researchers used hedges more frequently than English L1 researchers (9.67 *vs*. 7.04 per 1,000 words). Chinese L1 researchers preferred to use the self-mention phrase “this paper”, while English L1 researchers employed slightly more boosters and self-mentions generally. Attitude markers were found comparable between the two corpora (4.63 *vs*. 4.28 per 1,000 words), which can reflect the demands of this academic genre for objectivity. These usage patterns are consistent across all journal quartiles and are generally not influenced by impact levels of the journals. Further analysis of the subcategories shows that Chinese L1 researchers depended more on modal and phrase-based hedges, while English L1 ones used a more diversified range of hedging strategies. In terms of English L1 countries, researchers from the USA, the UK, and Canada shared similar rhetorical conventions. The findings indicate that cultural traditions and disciplinary norms jointly shape the use of stance markers. This study enriches cross-cultural academic discourse studies in hard sciences, and provides practical guidance on the academic English writing instruction for Chinese L1 researchers, especially graduate students.

## 1. Introduction

Academic discourse is a social communication act involving both writers and readers [1–3]. Against the backdrop of global scientific integration, publishing research findings in English has become a universal requirement for academic recognition and knowledge dissemination across all disciplines. It has become an essential practice for graduate students and researchers to publish academic papers written in English in many countries, especially in China where science and technology have advanced rapidly in the past two decades. Great institutional demands, high expectations from the academic community and writing conventions all make academic writing in English increasingly challenging and publishing research findings more competitive [4,5].

This is especially true for nonnative novice researchers, who face great challenges deriving from their limited English proficiency [6,7], their native language interference [4], as well as their unfamiliarity with the rhetorical conventions of academic discourse [4,8]. Specifically, nonnative writers often struggle to follow international academic norms due to cultural and linguistic differences and the lack of proficiency in using genre-specific linguistic markers [6,7]. Among these genre-specific skills, the appropriate use of stance-taking devices has been identified as one of the most challenging barriers for nonnative researchers, as it requires not only linguistic competence but also deep understanding of disciplinary and cultural rhetorical norms.

As a primary platform for academic communication, a research article abstract functions as a highly condensed summary of the academic work of the researchers and an important bridge for interdisciplinary knowledge exchange. The quality of an abstract crucially determines the readability and acceptability of an academic article, and thus shapes the visibility and impact of the article. Abstracts perform perhaps the most important function among all sections of a research article because they summarize the whole paper, help researchers obtain information, and attract readers to continue reading [9]. Its role has become increasingly prominent with rapid knowledge accumulation and growing interdisciplinary cooperation in modern society.

Effective abstract writing depends greatly on the strategic use of key linguistic devices, among which stance markers are particularly prominent. Stance markers allow researchers to convey their evaluations, express certainty or caution, and build a persuasive academic position [1], yet few studies have explored cross-disciplinary differences in their use. Embodying the stance of the authors on the research questions, methods, findings and possible implications, which are considered to be the key elements of an abstract, stance markers can facilitate the interaction between the authors and the readers from diverse backgrounds.

While the importance of stance markers is universally acknowledged, their appropriate use is complicated by significant disciplinary and cultural variations. A tough challenge for researchers in composing high-quality English abstracts lies in the fact that there are fundamental differences in academic discourse across disciplines, especially in the use of stance markers. Research indicates that disciplines influence the rhetorical choices of the researchers when they are writing their academic papers [11,12]. These different writing conventions across disciplines can cause difficulty in understanding academic discourse for the readers.

Besides cross-disciplinary obstacles, in abstract writing, researchers often face significant cross-cultural rhetorical challenges. Cultural elements can profoundly influence how authors employ stance markers to balance their authorial authority and proper modesty, potentially leading to different writing patterns between nonnatives and native English authors [10,11]. Such differences may be barriers to rapid academic dissemination globally. Overall, stance norms in abstract writing are shaped by two elements: disciplinary paradigms that can determine the acceptable degree and style of authorial stance [12], and cultural values that can influence the specific rhetorical choices in stance markers [1].

Existing cross-cultural comparative studies have extensively examined academic discourse (including soft disciplines and some hard sciences) and non-scientific discourse (*e.g*., news reports, editorials, and business writing); however, abstracts from hard disciplines such as Materials Science have rarely been investigated empirically. As a cornerstone of modern civilization and the backbone of global technological development, Materials Science is a quintessential hard discipline that prioritizes empirical evidence and imposes unique rhetorical demands for precision and objectivity on academic discourse. However, there remains a lack of systematic investigation into stance marker usage in this field. In addition, few studies have specifically focused on the research article abstract—a genre that serves as the first point of contact between authors and readers and has distinct rhetorical constraints compared to the full-length article. Most critically, no large-scale corpus-based study has yet compared the stance-taking practices of native Chinese-speaking (Chinese L1) and native English-speaking (English L1) researchers in Materials Science abstracts, creating a critical gap in both cross-cultural academic discourse theory and discipline-specific academic writing instruction.

Therefore, filling this gap carries substantial theoretical and practical value. Our study is significant not only for academic pragmatics research, but also for providing evidence-based guidance on academic writing instruction, especially for novice researchers in this critical field. By examining how stance markers are used, we can explore and identify both disciplinary conventions and cross-cultural expectations, and researchers in this field can learn to better meet the expectations of target readers when writing their academic abstracts, which will facilitate the dissemination of their research findings.

In this study we attempted to compare stance markers used in the abstracts of research articles in Materials Science written by Chinese and English L1 researchers. Our study aimed to identify the possible variations in their use, discuss these variation patterns within the context of discipline and culture, and offer possible implications for effective academic communication and writing instruction in this nationally and even globally significant field.

## 2. Literature review

### 2.1. Theoretical frameworks

It is widely agreed that stance analysis is commonly approached through two major theoretical frameworks. However, a persistent tension exists between formal-linguistic classification and functional-communicative interpretation.

Biber *et al*. [13] adopted a formal-linguistic approach, categorizing stance expressions into grammatical constructs (including modal verbs, stance adverbs, and complement clauses) and examining their distribution across registers. This framework is valued for its quantitative rigor [14], which enables cross-linguistic and diachronic comparisons. However, a critical limitation is its overemphasis on linguistic form rather than interactive communicative functions, which restricts its utility in exploring how writers construct relationships with readers and construct textual identity, the core concerns of stance research in Hyland’s work [1,2]. For a critical evaluation of the strengths and limitations of this formal-linguistic approach, see the review of the book *Longman Grammar of Spoken and Written English* by Gallagher (2000) [15].

In contrast, Hyland’s interactional metadiscourse model [1] addresses this limitation and aligns more closely with the communicative focus of contemporary stance research. This model has gained widespread acceptance due to its emphasis on writer-reader interaction and an empirically validated classification system. In this model, stance is defined as “writer-oriented strategies that construct a textual ‘voice’ or community-recognized personality” (*p*. 176), mediating the relationships among writers, readers, and propositions without introducing new propositional content. The model examines how writers negotiate their stance, construct their identity, and interact with potential readers through four core categories of stance markers: hedges (*e.g.*, may, possibly), boosters (*e.g.*, clearly, undoubtedly), attitude markers (*e.g.*, fortunately, crucial), and self-mentions (*e.g.*, I, we, this study) [1]. Its corpus-driven empirical design and clear functional categorization has made it widely applied [7,10,16], rendering it particularly suitable for the analysis of stance in academic discourse, where functional meaning and writer-reader interaction are paramount.

Hyland’s model is particularly valuable for analyzing stance in written academic discourse, as it offers an explicit, operable taxonomy. Based on this taxonomy, items can be reliably retrieved and quantified using corpus tools, and this approach, successfully adopted in a range of studies [17,18], has yielded evidence-based insights for academic writing instruction and assessment, thereby confirming the taxonomy’s practicality and reliability.

The framework also demonstrates strong applicability in comparative studies of newly emerging and traditional academic genres. For instance, a comparison between the Open Accessible Summaries in Language Studies (OASIS) and traditional research article abstracts found that both genres predominantly use hedges as the most frequent stance markers and directives as the most frequent engagement markers. However, OASIS summaries exhibit a significantly higher overall frequency of both stance and engagement markers compared to scientific abstracts, with a particularly notable increase in the use of attitude markers and questions [19]. This finding further validates the applicability of Hyland’s framework in cross-genre stance analysis and enriches the theoretical understanding that stance expression is dynamically adjusted according to potential readers and communicative purpose.

A cross-linguistic study of research article conclusions similarly demonstrates that Chinese and English writers follow comparable move structures but differ in stance choices: Chinese writers favor boosters and attitude markers, while English writers prefer hedges and self-mentions [20]. This confirms the effectiveness of Hyland’s model in cross-linguistic and cross-move analyses, and highlights that stance choices are jointly shaped by cultural-rhetorical traditions and discourse functions. Reported variations in stance marker use have also been associated with disciplinary norms, diachronic shifts, writers’ language proficiency, and genre-specific conventions.

### 2.2. Disciplinary variations and diachronic changes

There is a well-established consensus that stance marker usage varies systematically across academic fields and evolves diachronically, driven by disciplinary epistemologies and changing practices in academic communication.

The use of stance markers in academic texts exhibits significant cross-disciplinary variation [21], underscoring the necessity of our conducting stance research within clearly defined disciplinary boundaries to generate pedagogically meaningful insights. Such variations are especially evident in research article abstracts. In addition, stance marker use has been found to undergo diachronic shifts [22].

Diachronic studies on abstracts indicate significant changes in the use of stance markers, suggesting that abstract writing is not static. A 50-year diachronic analysis reveals that the distribution of stance features has shifted considerably across disciplines, with soft knowledge fields decreasing their use of hedges and boosters, while electrical engineering shows a dramatic increase in boosters to emphasize certainty [12]. Another corpus-based study further confirms that research article abstracts should be treated as a distinct genre [23]. The data demonstrates a specific distribution of interactional metadiscourse in abstracts: compared to full-length articles, abstracts make far more use of boosters and far less use of hedges [23]. These findings carry direct pedagogical implications for the targeted teaching of abstract writing.

A critical shortcoming of existing cross-disciplinary research, however, is that it has largely restricted itself to broad distinctions between hard and soft sciences, without investigating the specific stance conventions in applied engineering disciplines like Materials Science.

In a relevant cross-disciplinary investigation, Alghazo *et al*. [14] examined grammatical devices of stance in research article abstracts in applied linguistics and literature using Biber *et al*.’s framework. The study found that stance complement clauses were the most frequent stance marker in both disciplines, but significant differences existed in other devices. That study is particularly valuable because it highlights the need to explore stance-marking variations even between closely related sub-disciplines, thereby filling a gap left by previous cross-disciplinary research. This finding reinforces the need for discipline-specific investigations such as the current study on Materials Science.

Diachronic studies reveal that stance expression evolves with disciplinary practices and broader trends in academic communication. Gillaerts and Van de Velde’s diachronic analysis of applied linguistics abstracts documented a significant overall decline in interactional metadiscourse, characterized primarily by a reduction in boosters and attitude markers, though the use of hedges showed a relative increase [23]. Similarly, Hyland and Jiang [12] observed disciplinary variations in booster usage across four fields between 1965 and 2015: boosters increased in electrical engineering, as authors sought to ensure readers were aware of the strength of their results, but decreased in applied linguistics, sociology, and biology, indicating a shift toward more empirically oriented and author-absent writing.

However, this evolutionary pattern is discipline-specific and not uniform across academic fields. Academic writing in psychology has become more personal and confident [5], while medical writing has grown increasingly cautious [22]. Some traditional stance resources, specifically modal verbs, semi-modal verbs, adverbs, and adjectives that express uncertainty, have declined in frequency [24]. As a traditional stance resource, the frequency of the evaluative that structure has declined by about 20%, primarily because writers are shifting towards more economical lexico-grammatical options to accommodate the information explosion and a stylistic shift towards more compact expression in academic publishing [25]. This evolution is embedded within the conventions of abstract writing itself, where authors must strategically convey the value of their research within strict space constraints, skillfully balancing academic norms with personal perspectives through the strategic deployment of stance markers.

Against the backdrop of public health emergencies, stance expression in medical disciplines exhibits distinctive diachronic and genre-specific variation, thereby further enriching our understanding of both disciplinary and temporal differences in the use of stance markers. A study of medical journal abstracts and highlights on COVID-19 found that both these promotional academic genres make extensive use of attitude markers to underscore research value [26]. Furthermore, driven by their inherent promotional need to rapidly capture readers’ attention and by strict word limits, highlights exhibit a significantly higher density of attitude markers than abstracts [26]. This feature further contributes to the study of stance variation across sub-genres within hard disciplines.

In addition to disciplinary and situational variations, the emergence of AI-generated text has introduced a new dimension to diachronic and genre-based research on academic stance, bringing a novel research perspective to the existing literature. According to Zhang and Zhang [27], ChatGPT-generated abstracts use more attitude markers and implicit stance expressions than human-written abstracts, offering a novel human–AI contrast for disciplinary variation studies. This emerging research direction not only expands the scope of stance marker research but also provides fresh insights for guiding academic writing instruction in the context of intelligent technologies. According to the study of Ferrer (2024) [28] on metadiscourse markers in aeronautics and aerospace engineering research articles, interactive markers predominated over interactional markers, suggesting that writers in this field prioritize textual cohesion. Interestingly, the use of interactional resources revealed a degree of informality through the employment of self-mentions, a finding that contradicts traditional assumptions about impersonal writing in engineering fields. This suggests that even within the broader category of hard sciences, stance conventions may vary considerably across sub-fields, underscoring the importance of discipline-specific studies such as the present investigation of Materials Science.

Despite these advances, no diachronic or genre-specific stance research has yet focused on Materials Science, leaving the distinctive rhetorical evolution of this field unaddressed. Given that stance marker usage has undergone diachronic shifts, it is essential to use up-to-date corpus samples to accurately capture current norms and ensure that academic instruction remains current. This consideration motivates our decision to restrict the corpus to the 2023–2025 period.

### 2.3. English proficiency, cross-cultural perspectives and generic practice

Broad scholarly agreement confirms that stance expression is shaped by L2 English proficiency, cultural-rhetorical traditions, and genre-specific conventions, yet critical gaps remain in cross-cultural and hard-discipline empirical research.

The English proficiency and cultural background of writers profoundly influence their use of stance expressions and the effectiveness of their genre practice. Novice writers with English as L2 (L2 writers) tend to overuse boosters and underuse hedges, resulting in overly assertive, monologic stances, whereas advanced academic writing is characterized by a strategic balance that favors hedges to construct a measured, dialogic, and authoritative voice [17,29]. Higher-proficiency L2 writers demonstrate greater frequency, variety, and complexity in their use of stance nouns [30]. Thus, for L2 writers, appropriate deployment of stance expressions remains a central challenge in academic writing. They often differ from L1 speakers in the frequency and accuracy of self-mentions, struggling to balance the need to highlight personal contributions with the imperative to adhere to disciplinary norms [4]. L2 writers must not only acquire stance expressions, but also learn to deploy them flexibly and appropriately to meet communicative goals within the conventions of their disciplinary genres [31].

A study of doctoral engineering students also confirms that even trained L2 writers often exhibit a metacognitive gap regarding how stance operates in their citations [32]. Specifically, despite frequently deploying stance markers in their texts, these emerging scholars tend to perceive themselves as neutral reporters of knowledge [32]. This discordance between writers’ intentions and their linguistic choices highlights the critical need for explicit instruction and empirical benchmarks, precisely what the present corpus-based comparison aims to provide.

Beyond proficiency, cultural and rhetorical traditions also modulate stance expression. Individualistic and collectivistic cultural values give rise to distinct stance-taking practices: the letters of U.S. CEOs are found to favor boosters to project authority and confidence, while Chinese CEOs balance hedges with boosters to reflect norms of modesty and harmony [11]. In news commentaries, *The New York Times* uses more self-mentions and engagement markers to encourage reader interaction, while *China Daily* relies more heavily on stance markers to emphasize collective perspectives, mirroring cultural discourse norms [33].

Cross-cultural comparisons between English and Arabic provide further evidence. Alghazo *et al*. (2021) [18] compared social science abstracts and found that Arabic writers used fewer hedges but more boosters and attitude markers, and explicitly employed self-mentions; English writers, by contrast, adopted a more cautious stance, using more hedges, and avoided first-person pronouns. These differences can be attributed to specific disciplinary natures and traditional academic writing conventions: the frequent use of hedges in English abstracts stems from the nature of social sciences where precise answers are rare, while the absence of self-mentions reflects the traditional view of academic writing as an impersonal form of discourse that discourages personal pronouns. That study is methodologically close to the present research as it also focuses on research article abstracts and adopts the same analytical framework. A key limitation of this line of cross-cultural research, however, is that it has exclusively examined soft disciplines and English-Arabic comparisons, leaving untested the applicability of such patterns to hard sciences and Chinese-English comparisons.

Cross-linguistic differences are equally evident in persuasive genres such as editorials. Alghazo *et al*. (2024) [34] compared English and Arabic newspaper editorials and found that Arabic editorials predominantly employed attitude markers, followed by boosters, with fewer hedges; English editorials, in contrast, relied mainly on hedges and attitude markers, displaying an overall more tentative stance. This indicates that culturally inflected stance preferences extend beyond academic discourse into public argumentative genres, further confirming the culture-specific nature of stance expression.

Genre-specific rhetorical traditions significantly modulate stance. In 3MT (Three-Minute Thesis) presentations, hard-science presenters deploy more attitude markers to highlight research significance for public outreach, while soft-science presenters use more questions to engage broad audiences [21]. Book reviews on academic blogs, influenced by the conventions of digital rhetoric, contain more stance markers (especially hedges, boosters and self-mentions) than journal reviews, as bloggers balance persuasion with openness to reader feedback [35]. In informational-promotional discourse, English texts rely on self-mentions to build ethos, while Spanish texts employ inclusive *we* to emphasize logos, reflecting genre-specific rhetorical norms [36]. Similarly, in Agricultural Science, writers use the fewest hedges in Methods but significantly more in Discussions and Conclusions to mitigate research claims [37]. These findings underscore the need for L2 writers, including Chinese researchers writing in English, to be cognizant of discipline-specific rhetorical conventions and intrageneric practices in their academic writing. One variable that remains underexplored, however, is journal impact level, which makes it impossible to ascertain whether journal prestige moderates the influence of cultural and disciplinary factors on stance usage.

It is widely acknowledged that stance expression is not merely linguistic but deeply embedded in cultural values and rhetorical conventions. Such differences pose distinct challenges in research abstract writing—a core medium of academic communication, where writers must accurately convey research value within a limited space while simultaneously balancing disciplinary norms and authorial stance. Flexible use of stance expressions is therefore essential. Writers must assume a dual role: representing their research and evaluating their findings. The construction of such a discoursal scholarly identity has been central to research writing [38]. For L2 writers, particularly Chinese authors writing in English, mastering stance expressions in discipline-specific genres like Materials Science abstracts requires not only linguistic knowledge but also heightened awareness of cross-cultural and cross-generic rhetorical expectations.

Collectively, these unexplored aspects result in an absence of discipline-specific guidance for Chinese Materials Science researchers writing English abstracts, a significant practical shortfall in academic writing instruction.

### 2.4. Research questions

Based on the synthesis of the preceding literature, three core, interconnected research gaps are identified, which directly justify and guide the present study.

Past research has confirmed disciplinary and cross-cultural variations in stance expressions, yet critical gaps remain regarding Materials Science abstracts. While cross-disciplinary studies have broadly contrasted hard and soft sciences, the specific stance conventions of Materials Science—an applied engineering discipline—remain underexplored. Second, although cross-linguistic comparisons exist (*e.g.*, English *vs*. Arabic), no study has systematically compared Chinese and English L1 authors’ use of stance expressions in Materials Science abstracts. Furthermore, most existing corpus-based stance studies have used samples without distinguishing journal impact levels. It therefore remains unknown whether journal quartiles influence stance usage—for instance, whether high-tier journals enforce a more cautious style, or whether cross-cultural differences vary by publication tier. This study addresses these gaps by constructing controlled corpora of Materials Science research article abstracts from journals across all four quartile rankings in the Journal Citation Reports, thereby enabling a robust comparison that disentangles author L1 background from journal quality norms.

To address the practical need for discipline-specific academic writing instruction for graduate students and professional researchers in China, this study focuses on Materials Science research article abstracts. It systematically compares Chinese and English L1 authors in their use of stance expressions (hedges, boosters, attitude markers, self-mentions), with attention to their frequency, diversity, and the potential influence of journal impact level.

This study is guided by the following research questions:

1. To what extent do Chinese and English L1 authors differ in the frequency and type diversity of stance expressions in Materials Science abstracts when controlling for journal quartile?
2. How do cultural-rhetorical traditions (*e.g.*, collectivism *vs*. individualism) and disciplinary norms (*e.g.*, emphasis on empirical caution) jointly shape the observed cross-cultural differences, and do these differences remain consistent across journal impact levels?

## 3. Methods

### 3.1. Corpus construction and data selection

The corpora were constructed using abstracts of 1002 research articles in the field of Materials Science, indexed in the Web of Science Core Collection and published between 2023 and 2025 (Appendix A). Half of these articles were first authored by the researchers affiliated with the institutions in mainland China and the other half were first authored by the researchers affiliated with the institutions in English-dominant academic contexts. Review articles, short communications, and editorials were excluded. Journals were selected and stratified into four groups (Q1 to Q4) based on their corresponding quartile rankings in the Journal Citation Reports. We employed this stratification to investigate whether the use of stance markers differed systematically across journals of varying academic prestige and rhetorical constraints. For each quartile, abstracts were sampled from two types of author groups. The first corpus, operationally labeled as the Chinese L1 corpus, consisted of the abstracts whose first authors were affiliated with the institutions located in mainland China. The second corpus, operationally labeled as the English L1 corpus, consisted of abstracts whose first authors were affiliated with institutions located in English-dominant academic contexts, including the USA, the UK, Canada, Australia, New Zealand, Singapore, and Ireland. In each quartile, the number of articles by Chinese L1 authors is identical to that by English L1 authors, with per-group counts of 64 (Q1), 142 (Q2), 152 (Q3), and 143 (Q4), respectively (Appendix A).

Author grouping was determined mainly based on the institutional affiliations of the first authors at the time of publication. Author names were used only as supplementary information during manual screening and were not treated as the primary criterion for classification. In this study, the terms “Chinese L1” and “English L1” are used as operational corpus labels. They do not indicate that the actual first language, nationality, or educational history of every author was independently verified. To reduce possible classification errors, we further conducted a manual check on a random sample of 25 abstracts, approximately 5% of the total corpus, by referring to available author biographies, acknowledgments, and institutional webpages. No clear cases requiring reclassification were identified. Nevertheless, possible boundary cases, such as internationally mobile or multilingual scholars, are acknowledged as a limitation of the study.

Singapore was included in the English L1 corpus because English plays a predominant role in its higher education, and the country’s academic publishing system closely aligns with international English-language academic norms. Considering its multilingual sociolinguistic context, a sensitivity analysis excluding Singapore-affiliated abstracts was also conducted to examine whether their inclusion affected the overall findings.

To ensure comparability across the journal quartiles, we selected a fixed number of abstracts for each corpus within each quartile. The final samples therefore constituted two balanced corpora across journal ranks and author groups, allowing systematic comparisons both overall and within quartile-specific contexts.

### 3.2. Identification of stance markers

Stance markers were identified following Hyland’s interactional framework [1], which classifies stance expressions into four categories: hedges, boosters, attitude markers, and self-mentions. We adopted a lexicon-based approach to identify these stance expressions in the abstracts. The initial word lists were compiled following Hyland (2005) [1] classification and were further refined to better capture stance expressions commonly used in the selected abstracts. Automated scripts were used to retrieve candidate stance markers from the corpora. To ensure consistency, all candidate items were manually checked in context, and ambiguous cases were discussed among the researchers and resolved by consensus. Since the study adopted a lexicon-based design rather than open-ended manual annotation, manual checking focused mainly on applying predefined coding rules consistently and reducing false positives, especially for potentially ambiguous items. The frequencies of four core stance markers, *i.e.,* hedges, boosters, attitude markers, and self-mentions, were calculated by dividing the count of each marker by the total number of words in each abstract. The resulting per-abstract raw frequencies in each corpus were then normalized to a mean per 1,000 words to facilitate cross-corpus comparison.

### 3.3. Statistical analysis

Statistical analyses were conducted to compare stance marker usage between Chinese and English L1 authors across the two corpora and within journal quartiles. Normality assumptions were examined prior to inferential testing. As several variables did not follow a normal distribution, nonparametric Mann–Whitney *U* tests were applied for all group-level comparisons of normalized frequencies. Bonferroni correction was used where multiple comparisons were conducted to control the risk of Type I error inflation. All analyses were performed at the abstract level. Stance items with extremely low frequencies (*e.g*., “us”) were excluded from inferential statistical testing and reported descriptively only. Effect sizes (*r*), calculated as |*Z*|/$\sqrt{N}$ with 0.1, 0.3 and 0.5 defined as small, medium and large effect thresholds respectively, were reported alongside *p* values to reflect the magnitude of observed differences, where *Z* refers to the standardized *Z*-scores from the Mann–Whitney *U* tests and *N* denotes the total number of abstracts in the comparisons. A sensitivity analysis excluding Singapore-affiliated abstracts was also conducted to examine whether the inclusion of Singapore affected the overall findings. In addition, negative binomial regression models were fitted as a robustness check for raw count data, with author group and journal quartile entered as predictors and abstract word count entered as an offset.

## 4. Results and discussion

### 4.1. Overall stance usage in the two corpora

The Chinese and English L1 corpora have 94,837 and 101,076 words, respectively (Table 1). The data reveal that the most evident difference between the two corpora lies in the use of hedges, while boosters, attitude markers, and self-mentions show relatively comparable overall patterns. In absolute terms, abstracts in the Chinese L1 corpus exhibited a higher frequency of hedges, with 919 instances documented, compared to 716 instances in the English L1 corpus. When frequencies were normalized to per 1,000 words, hedges remained more frequent in the Chinese L1 corpus, with a mean rate of 9.67 per 1,000 words, relative to 7.04 in the English L1 corpus (Table 1). The Mann–Whitney *U* test further showed that this difference remained significant after Bonferroni correction (*p* < 0.001), although the effect size was small (*r* = 0.159).

**Table 1:** Overall stance usage in the two corpora.

Corpus	Number of Abstracts	Total words	Hedges	Boosters	Attitude markers	Self-mentions
			(Count/normalized mean per 1,000 words)			
Chinese L1	501	94,837	919/9.67	680/7.34	447/4.63	871/10.06
English L1	501	101,076	716/7.04	719/7.17	445/4.28	884/9.18

In contrast, boosters, attitude markers, and total self-mentions did not show significant group differences after Bonferroni correction (*p* > 0.05). Boosters occurred at similar normalized frequencies in the two corpora, with mean values of 7.34 in the Chinese L1 corpus and 7.17 in the English L1 corpus. Attitude markers were also comparable, with normalized mean frequencies of 4.63 and 4.28 per 1,000 words in the Chinese and English L1 corpora, respectively (Table 1). Total self-mentions showed a small numerical difference, with 10.06 per 1,000 words in the Chinese L1 corpus and 9.18 in the English L1 corpus (Table 1), but this difference was not statistically significant (*p* > 0.05).

The negative binomial regression robustness check yielded the same overall pattern, with hedges remaining the only major stance category showing a significant group difference. These results indicate that the two corpora exhibit broadly similar patterns across most major stance categories, with hedging emerging as the clearest point of divergence. They further suggest that both Chinese and English L1 researchers remained relatively restrained in expressing evaluative or emotional stance, which aligns with the objectivity expected in academic texts. This pattern echoes Hyland’s cross-disciplinary finding that attitude markers are used sparingly in academic abstracts to preserve objectivity, regardless of cultural background [39].

### 4.2. Stance variations across journal quartiles

To determine whether differences in the use of stance markers varied by publication prestige, we further analyzed the data according to journal quartiles. Table 2 summarizes stance usage across Q1–Q4 journals.

**Table 2:** Stance usage across quartiles and groups (values are means per 1,000 words).

Quartile	Group	Hedges	Boosters	Attitude markers	Self-mentions
Q1	Chinese L1	8.87	6.07	4.80	5.96
	English L1	6.20	7.10	4.06	8.07
Q2	Chinese L1	9.68	6.97	4.02	5.86
	English L1	6.45	7.35	4.82	9.06
Q3	Chinese L1	8.66	6.60	6.83	9.35
	English L1	6.84	6.46	4.06	9.95
Q4	Chinese L1	11.09	9.07	2.81	16.80
	English L1	8.21	7.78	4.08	8.97

In terms of hedges, Chinese L1 researchers used them at higher frequencies than English L1 researchers across all journal quartiles. However, after Bonferroni correction, the difference was significant only in Q2 and Q4 journals, indicating that the overall difference in hedging was not equally strong across all journal ranks.

The use of boosters remained relatively stable across quartiles. Although numerical differences were observed between the two corpora, none of the group differences in boosters reached statistical significance after Bonferroni correction. This finding suggests that the two groups employed broadly similar strategies in expressing certainty across different journal quartiles.

Attitude markers showed a more fluctuating pattern. Chinese L1 researchers used more attitude markers in Q3 journals, whereas English L1 researchers showed a higher frequency in Q4 journals. Self-mentions also displayed inconsistent quartile-specific patterns, with English L1 researchers using more self-mentions in Q2 journals and Chinese L1 researchers using more self-mentions in Q4 journals. These results indicate that journal quartile influenced stance use in a category-specific rather than linear manner.

### 4.3. Internal distribution of stance subcategories

Besides overall frequencies, differences were also identified in stance subcategories. Table 3 shows the distribution of selected subcategories of hedges and boosters. Within the hedging subcategory, modal hedges and phrase-based hedges were two important resources in the Chinese L1 corpus. In particular, phrase-based hedges occurred more frequently in the Chinese L1 corpus than in the English L1 corpus, suggesting a relatively stronger use of formulaic expressions in presenting uncertainty. Modal hedges were also descriptively more frequent in the Chinese L1 corpus, whereas verbal hedges occurred at comparable levels in the two corpora. These findings suggest that the higher overall frequency of hedges in the Chinese L1 corpus was not evenly distributed across all hedge subcategories, but was especially reflected in phrase-based hedges.

**Table 3:** Internal distribution of selected subcategories of hedges and boosters (values are the total counts per item).

Corpus	Modal hedges	Verbal hedges	Phrase-based hedges	Assertive verbs	Emphatic adverbs
Chinese L1	410	149	185	615	15
English L1	334	152	83	667	11

It has been argued that in the academic writing of L2 learners, the expression of stance is often influenced by writing experience, academic conventions, and rhetorical awareness [7,40,41]. Similarly, the greater use of phrase-based hedges in the Chinese L1 corpus may indicate a preference for more explicit and recognizable hedging patterns. However, this pattern should be interpreted as a distributional tendency rather than direct evidence of psychological caution or cultural modesty. Such a tendency may be related to multiple factors, including academic writing training, institutional writing conventions, disciplinary expectations, and broader cultural-rhetorical traditions. Therefore, for nonnative researchers, being aware of academic conventions is essential. Researchers should be encouraged to use diverse hedging strategies to enhance the persuasive power of their academic work.

Boosters in the two corpora generally show slight variation: 7.34 per 1,000 words for Chinese L1 researchers and 7.17 for English L1 ones (Table 1). It shows both groups recognize the rhetorical value of boosters in asserting research validity. The data in Table 3 also reveal a common rhetorical emphasis across the two groups on assertive verbs rather than emphatic adverbs.

This pattern is consistent with the disciplinary preference for objective presentation in Materials Science. Although the total number of assertive verbs was slightly higher in the English L1 corpus, the overall use of boosters remained highly comparable between the two corpora. This finding indicates that the two groups are similar in strengthening their claims primarily through evidence-framing verbs, while they avoid more subjective forms of emphasis to conform to the established academic conventions.

Our findings echo the study by Afzaal et al. [6], which highlights that managing a diverse range of stance markers is a key challenge for EFL learners compared to native speakers. While native writers optimize stance markers for rhetorical effect and disciplinary conventions, non-native writers often exhibit distinct patterns, such as the overuse of self-mentions by novices or modal hedges by upper-intermediate learners. This underscores the necessity for explicit instruction to help L2 learners develop a more nuanced and audience-aware academic writing persona.

Table 4 summarizes the sub-category distribution of self-mentions in the two corpora. Overall, the two corpora demonstrate highly similar patterns in most self-mention subcategories. The uses of first-person plural pronouns (we, our, us), the phrase “this study”, and the total self-mention frequency show no statistically significant differences after Bonferroni correction, with effect sizes negligibly small. This similarity between the two groups indicates that many self-mention devices used in the construction of authorial presence have become relatively standardized across global academic discourse.

**Table 4:** Sub-categories of self-mentions in the corpora (*p* values are Bonferroni-adjusted).

	Chinese L1	English L1	*p*	*r*
	(Mean per 1,000 words)			
we	4.58	4.54	1.000	0.024
our	1.18	1.11	1.000	0.006
us	0.04	0.04	1.000	0.000
this study	2.12	2.22	1.000	0.009
this paper	1.42	0.49	<0.001	0.103

A key distinction lies in the use of the phrase “this paper”, which was used significantly more frequently by Chinese L1 researchers (M = 1.42) than by English L1 researchers (M = 0.49; adjusted *p* < 0.001). This preference for “this paper” reflects a tendency to position the text itself as the primary agent of the research rather than the researcher. This finding agrees with Zhao’s [10] observation that Chinese L1 scholars favor inanimate noun phrases like “this paper” to objectify their research. Nevertheless, the difference should be interpreted cautiously, as the present corpus data cannot directly establish the cultural or psychological motivations behind this choice.

This rhetorical choice may be related to varying academic writing conventions and pedagogical traditions. Chen [42] noted that the use of “this paper” may align with writing norms that discourage overt authorial prominence. By shifting the agent of the research to the text, writers can meet the need for self-reference while maintaining a relatively impersonal tone. Similarly, Can and Cangır [4] reported that nonnative scholars relied more on inanimate markers than British peers, reinforcing that objectified self-reference is common among some nonnative academic writers.

The two corpora demonstrate shared patterns in many stance subcategories, while differences appear in several specific forms. Phrase-based hedges and the phrase “this paper” were the most notable subcategory-level distinctions in the Chinese L1 corpus. These findings suggest that academic writing instruction should not simply advise writers to use more or fewer stance markers in general. Instead, more attention should be paid to the selection of specific stance resources, such as how to diversify hedging expressions and how to choose among “we”, “this study”, and “this paper” according to rhetorical purpose and journal expectations.

### 4.4. Variation within the English L1 corpus

Based on the data presented in Table 5, which details stance marker usage across several major English L1 countries, we observe both a shared pattern and some variation in rhetorical emphasis among the researchers from these countries. Since the sizes of the subcorpora were uneven across countries, this section is reported descriptively only, and no inferential claims are made about national differences.

**Table 5:** Descriptive stance usage across English L1 countries (values are the total counts per item).

Country	Hedges	Boosters	Attitude markers	Self-mentions	Total words
USA	426	451	280	610	66,001
UK	56	97	54	94	12,586
Canada	42	78	47	76	9,820
Australia	39	53	32	50	7,208
New Zealand	13	8	8	12	963
Singapore	25	21	17	34	3,491
Ireland	7	11	7	8	1,007

Overall, a broadly similar stance profile can be observed across these English L1 contexts. When frequencies are considered in relation to the total word counts, self-mentions and boosters appear to be relatively frequent categories, while attitude markers remain the least frequent category in most countries. This pattern suggests that researchers in these English L1 contexts generally construct authorial presence and express certainty, while using explicit evaluative expressions more selectively. Such a tendency is also consistent with the objectivity-oriented rhetorical expectations of Materials Science abstracts.

Some descriptive differences can also be observed. The USA, with the largest number of words in this subcorpus, naturally accounts for the largest number of stance markers. The UK and Canada show broadly comparable distributions, whereas Australia also follows a similar general pattern. Singapore shows a comparable profile, but its results should be interpreted cautiously because of its multilingual sociolinguistic context. The samples from New Zealand and Ireland are much smaller, so their distributions should not be overinterpreted as stable national patterns.

These findings suggest that the English L1 corpus is not entirely homogeneous, but the observed variation should be understood as descriptive rather than inferential. Given the uneven corpus sizes across countries, especially the small samples from New Zealand and Ireland, this analysis mainly serves to show the internal composition of the English L1 corpus. The sensitivity analysis excluding Singapore-affiliated abstracts further indicated that the inclusion of Singapore did not materially change the overall findings.

## 5. Conclusion

Our study has revealed a nuanced pattern in the use of stance markers in research article abstracts between Chinese and English L1 researchers in the field of Materials Science. While both groups draw upon a fundamentally shared repertoire of stance resources, the most robust overall difference lies in the use of hedges. The Chinese L1 corpus used hedges more frequently than the English L1 corpus, although the effect size was small. By contrast, boosters, attitude markers, and total self-mentions did not show significant overall differences after Bonferroni correction. These findings suggest that the two corpora share broadly comparable stance practices in most major categories, with hedging being the clearest point of contrast.

The quartile-specific analysis further shows that these differences are not uniform across journal ranks. Q1 journals showed no significant group differences in the four main stance categories, suggesting stronger convergence in higher-ranked journals. The hedge difference was mainly evident in Q2 and Q4 journals, whereas attitude markers and self-mentions displayed more context-dependent patterns. At the subcategory level, phrase-based hedges and the self-mention phrase “this paper” were particularly notable in distinguishing the Chinese L1 corpus from the English L1 corpus. These results indicate that cultural-rhetorical traditions, disciplinary norms and journal contexts may jointly shape stance expression, but their influence is selective rather than uniform.

The findings also carry implications for academic English writing instruction in China. Instruction should not simply advise writers to use more or fewer stance markers in general. Instead, it should provide more explicit, corpus-informed guidance on the selection of specific stance resources, such as how to diversify hedging expressions and how to choose among “we”, “this study”, and “this paper” according to rhetorical purpose and journal expectations. Meanwhile, the cross-group consistency of boosters and attitude markers indicates that these categories may require less targeted intervention than is needed for hedging strategies and specific self-mention choices.

However, several limitations in our study must be acknowledged. First, in the annotating process of stance markers, some polysemous words such as “certain” were not functionally distinguished. All instances of “certain” were coded as boosters, including those with the adjectival meaning “specific” (*e.g*., “certain classes”). A pilot analysis of 100 abstracts (which were not used in this study) suggests that almost all instances of “certain” served as boosters; nevertheless, categorizing all occurrences without differentiating in detail might have led to a minor deviation in booster frequency and affected the absolute statistical accuracy. Second, the classification of authors as “Chinese L1” or “English L1” was mainly based on the institutional affiliation of the first author, with author names used only as supplementary information during manual screening. This strategy improves transparency and reproducibility but cannot fully capture the exact cultural, linguistic or educational backgrounds of all individual authors. Possible boundary cases, such as internationally mobile or multilingual scholars, may therefore affect the internal homogeneity of the two subcorpora. However, the additional manual check on 25 randomly sampled abstracts did not identify clear cases requiring reclassification, and the sensitivity analysis excluding Singapore-affiliated abstracts yielded the same overall pattern. Future research could further refine author classification by incorporating richer sociolinguistic metadata, such as authors’ educational trajectories and self-reported language backgrounds.

Drawing on these findings and limitations, we propose several directions for future research. First, expanding the scope of analysis from abstracts to full-length manuscripts could help reveal how stance markers are strategically distributed across different sections and whether rhetorical norms vary across sub-genres within the discipline. Second, adopting mixed methods is crucial. Supplementing corpus analysis with interviews could help uncover the motivations, perceptions and strategic choices of researchers behind the observed linguistic patterns. Third, the role of external factors also deserves investigation. A comprehensive analysis of journal guidelines and peer-review comments may reveal how editorial policies and publication processes influence the stance-taking practices of researchers.

## Supporting information

S1 Appendix A Supporting Information.zip. The file contains the full dataset of this study, including corpus metadata, frequencies disaggregated by Journal Citation Reports quartile (Q1–Q4) and author L1 group (Chinese vs. English L1), multi-level descriptive statistics, subcategory summaries by L1 group and English-speaking country, and results of between-group statistical tests.
(ZIP)
